# Supplementary material for: Augmentation of Bri2 molecular chaperone activity against amyloid-β reduces neurotoxicity in mouse hippocampus in vitro
Source: Commun Biol. 2020 Jan 20;3:32. doi: 10.1038/s42003-020-0757-z (PMC6971075; doi:10.1038/s42003-020-0757-z)
Supplement: Supplementary file 1 — Supplementary Information [file 42003_2020_757_MOESM1_ESM.pdf]

Supplementary figures

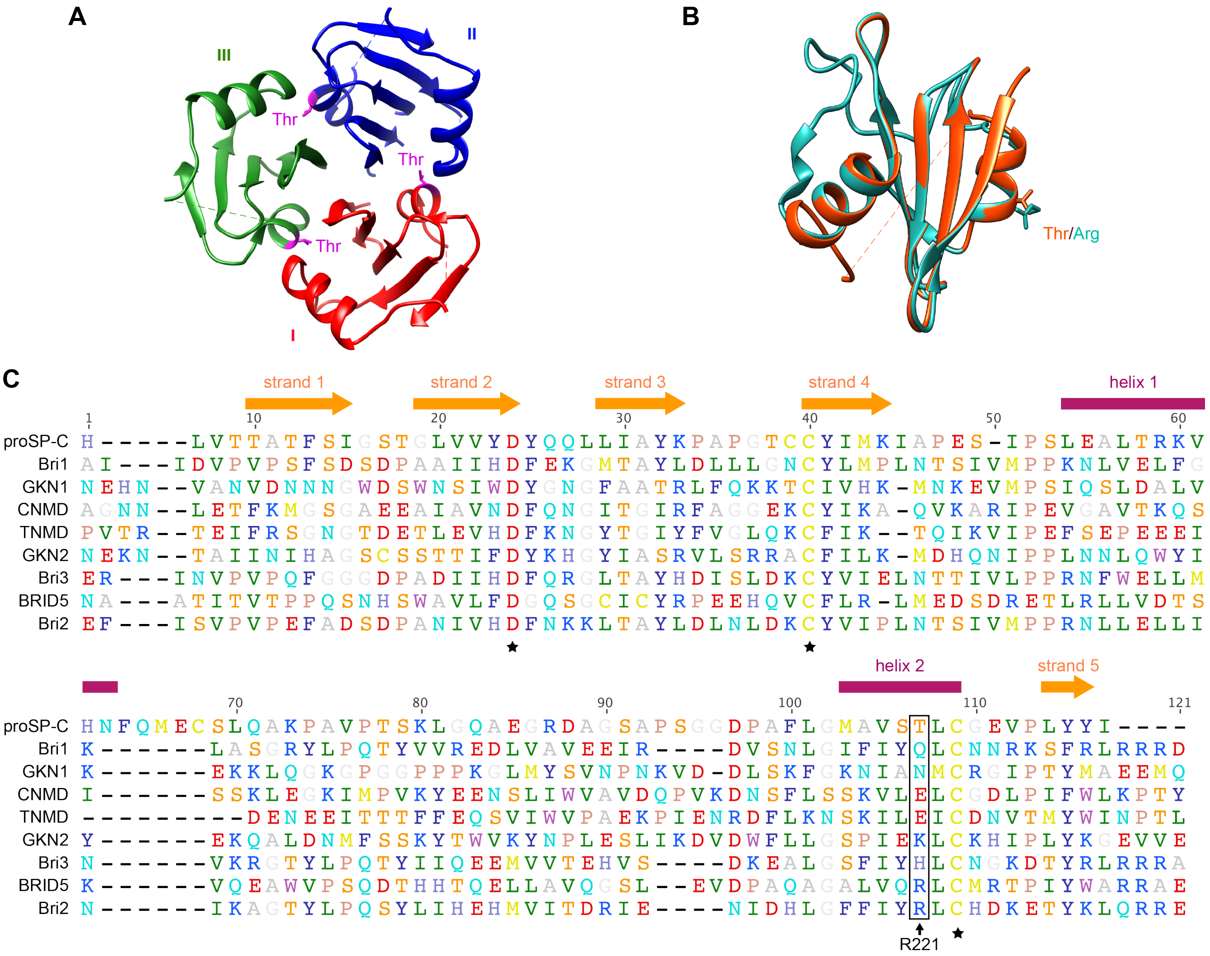

Supplementary Figure 1. Bri2 BRICHOS structure model and sequence alignment of human BRICHOS domains. (**A**) The crystal structure of rh proSP-C BRICHOS (PDB accession number: 2YAD) shows a homotrimer in which a Thr side-chain (identified in all three subunits) in helix 2 of one subunit points into a pocket of the neighbouring subunit. (**B**) Homology model of Bri2 BRICHOS (cyan) based on the proSP-C structure (orange[34]). The side-chain of Thr in proSP-C BRICHOS that corresponds to R221 in Bri2 BRICHOS is identified. (**C**) Amino acid sequences of BRICHOS domains aligned using ClustalW embedded in Geneious. Amino acid residues are color coded according to properties. Gaps (–) have been inserted in order to optimize residue identities. The position corresponding to R221 in Bri2 BRICHOS is boxed. Stars indicate the strictly conserved Asp (D) and Cys (C). The secondary structure elements given above the sequences are derived from the rh proSP-C BRICHOS crystal structure shown in **(A)**. The sequences shown are: proSP-C, pulmonary surfactant-associated protein C isoform 1 precursor [Homo sapiens] (pos. 87-197, GenBank accession no. NP_003009); Bri1, integral membrane protein 2A [Homo sapiens] (pos. 124-08, GenBank accession no. BAD97047); GKN1, KFTI489 [Homo sapiens] (pos. 44-154,

GenBank accession no. AAQ89409); CNMD, leukocyte cell-derived chemotaxin 1 isoform 2 precursor [Homo sapiens] (pos. 94-205, GenBank accession no. NP_001011705); TNMD, tenomodulin, isoform CRA_c [Homo sapiens] (pos. 83-190, GenBank accession no. EAX02808); GKN2 BRICHOS, VLTI465 [Homo sapiens] (pos. 36-147, GenBank accession no. AAQ89027); Bri3, Integral membrane protein 2C [Homo sapiens] (pos. 127-234, GenBank accession no. AAH02424); BRID5, hypothetical protein [Homo sapiens] (pos. 89-197, GenBank accession no. AAT09004); Bri2, integral membrane protein 2B [Homo sapiens] (pos. 128-235, GenBank accession no. NP_068839).

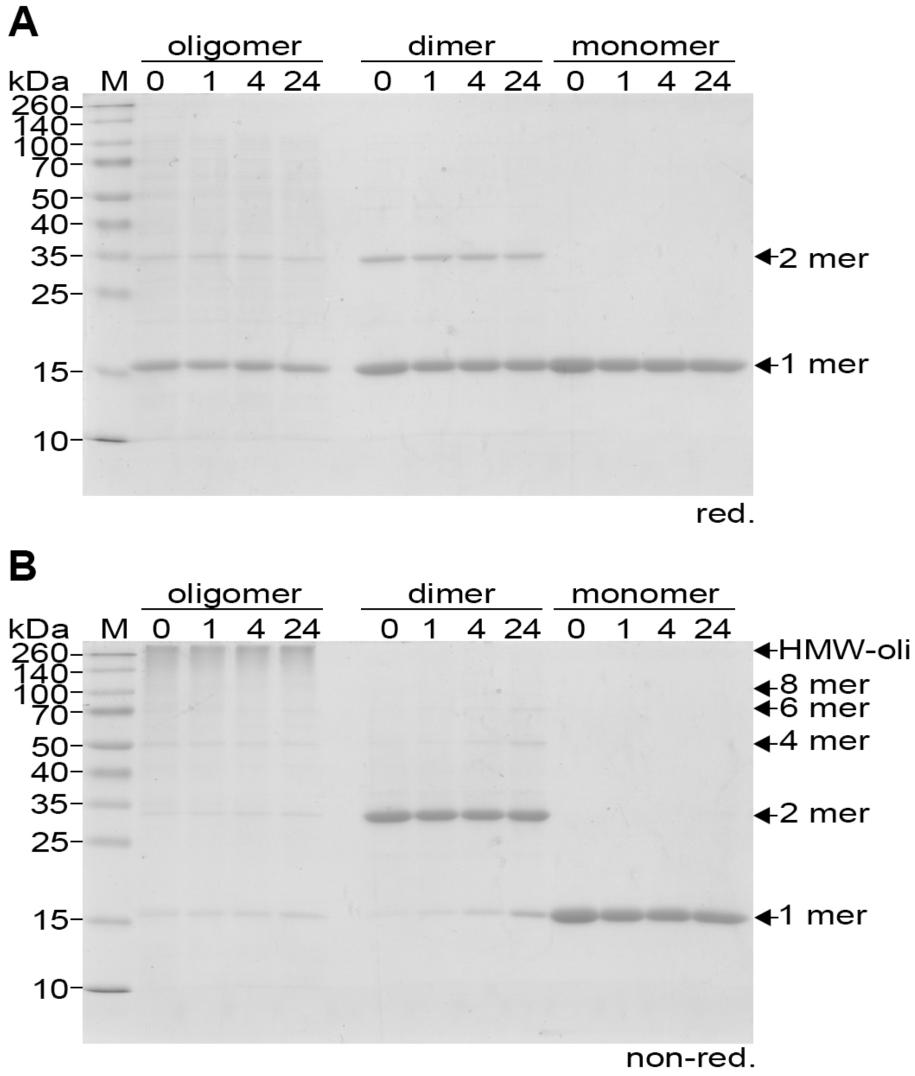

Supplementary Figure 2. Disulfide patterns and stabilities of rh Bri2 BRICHOS R221E species. SDS-PAGE analyses of SEC isolated rh Bri2 BRICHOS R221E species under reducing **(A)** and non-reducing **(B)** conditions without incubation (0 h) and after incubation in 20 mM NaPi pH 8.0 at 37°C for different time periods (hours) given above each lane.

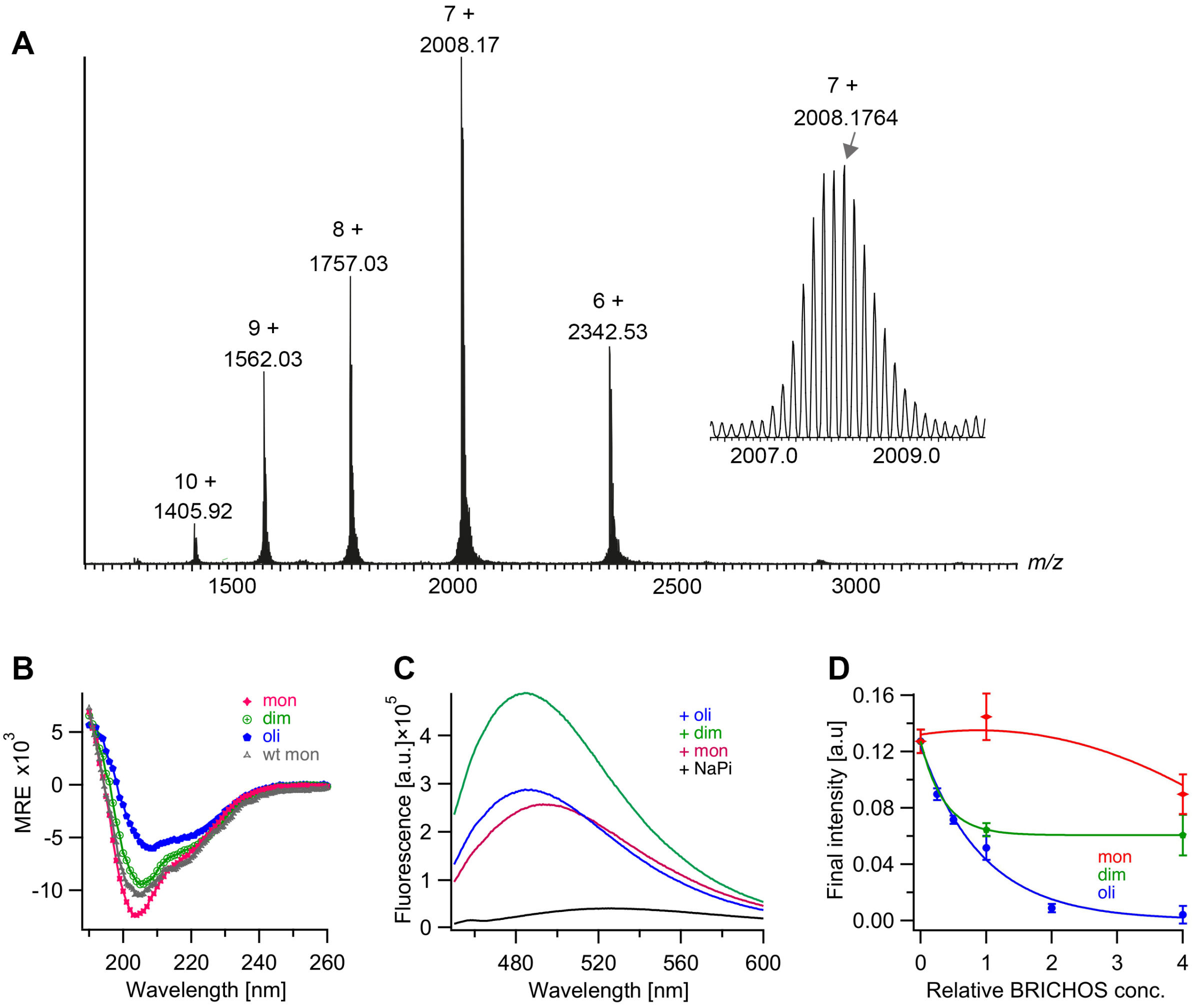

Supplementary Figure 3. Characterization of rh Bri2 BRICHOS R221E species. (**A**) ESI-MS spectrum of rh Bri2 BRICHOS R221E monomers. M/Z values and number of charges are given above each peak. The inset shows the zoomed in 7+ peak. (**B**) CD spectra of rh Bri2 BRICHOS R221E monomers, dimers and oligomers and rh wildtype (wt) Bri2 BRICHOS monomers. MRE is the mean molar residual ellipticity in deg·cm^2·dmol^{-1}. (**C**) Fluorescence emission of 10 µM bis-ANS in sodium phosphate buffer (black) or in the presence of 2 µM rh Bri2 BRICHOS R221E oligomer (blue), dimer (green), or monomer (red). (**D**) Effects of different molar ratios (referring to monomeric subunits) of rh Bri2 BRICHOS R221E oligomers (blue), dimers (green) and monomers (red) on aggregation of heat denatured citrate synthase (CS). The absorbance intensity at 360 nm after 1 h incubation of CS at 45°C is plotted *vs* the relative ratio of rh Bri2 BRICHOS species compared to CS. The data are presented as means ± standard deviations of 3–4 replicates.

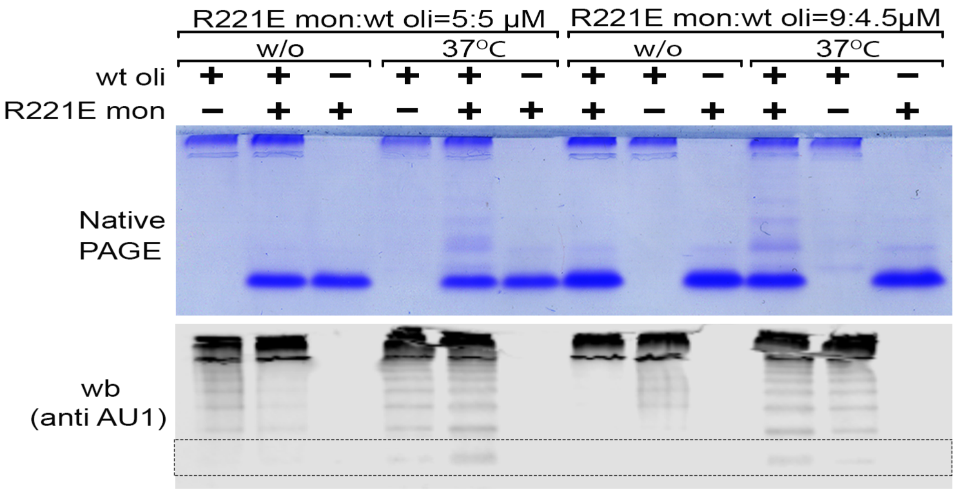

Supplementary Figure 4. Effects of coincubation of rh Bri2 BRICHOS R221E monomers and rh wildtype Bri2 BRICHOS oligomers. Rh Bri2 BRICHOS R221E monomers and rh wildtype (wt) Bri2 BRICHOS oligomers containing an AU1 tag (5:5 µM and 9:4.5 µM) were analysed without incubation (labelled w/o) or co-incubated at 37°C overnight, and analysed by native PAGE and western blot with an anti AU1 tag antibody. The dashed box indicates migration of monomers. Parts of the gel are also shown in **Figure 2E**.

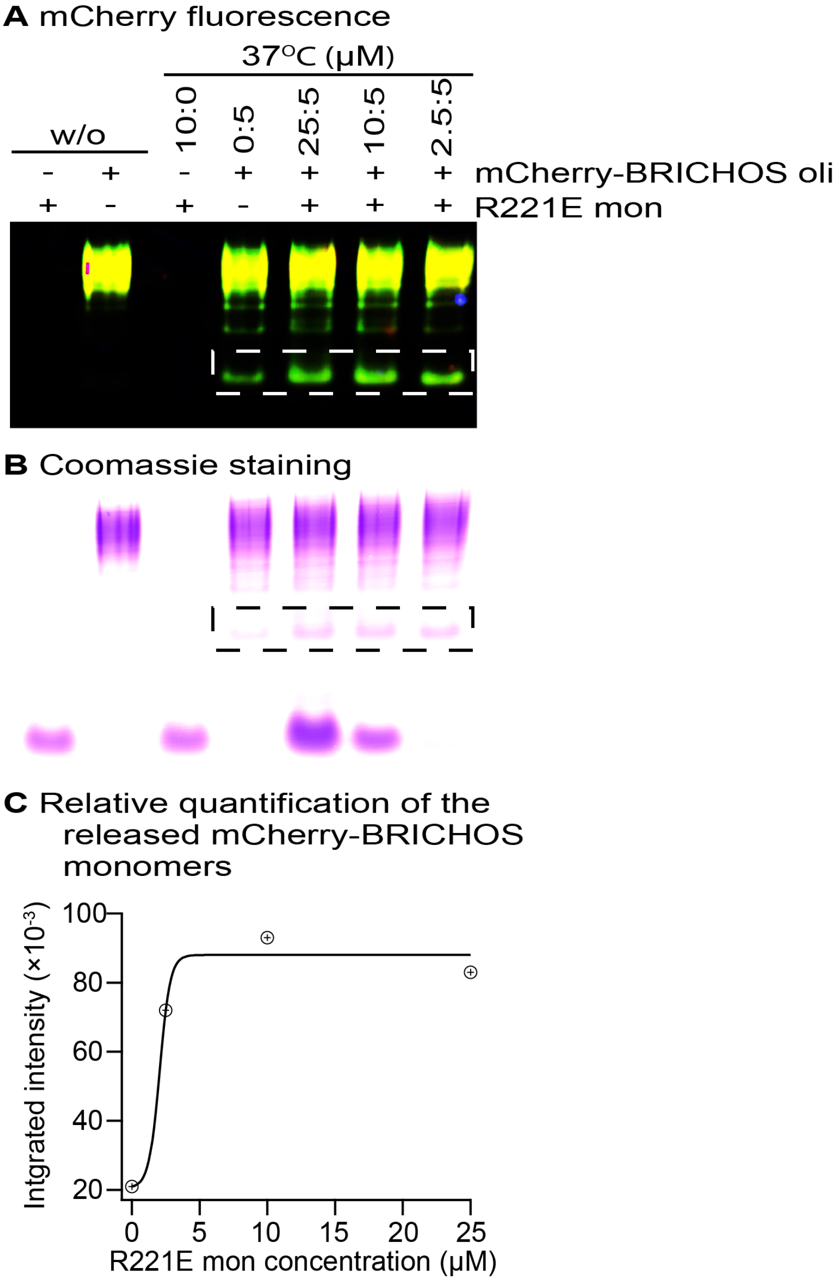

Supplementary Figure 5. Effects of coincubation of rh Bri2 BRICHOS R221E monomers and rh Bri2 BRICHOS-mCherry oligomers. Rh Bri2 BRICHOS R221E monomers and rh Bri2 BRICHOS-mCherry oligomers at different ratio were analysed without incubation (w/o) or co-incubated at 37°C overnight, and analysed by mCherry fluorescence (**A**) and Coomassie staining (**B**). The band intensities in the dashed box, corresponding to released monomers were evaluated by ImageJ and plotted as a function of mutant monomer concentration (**C**).

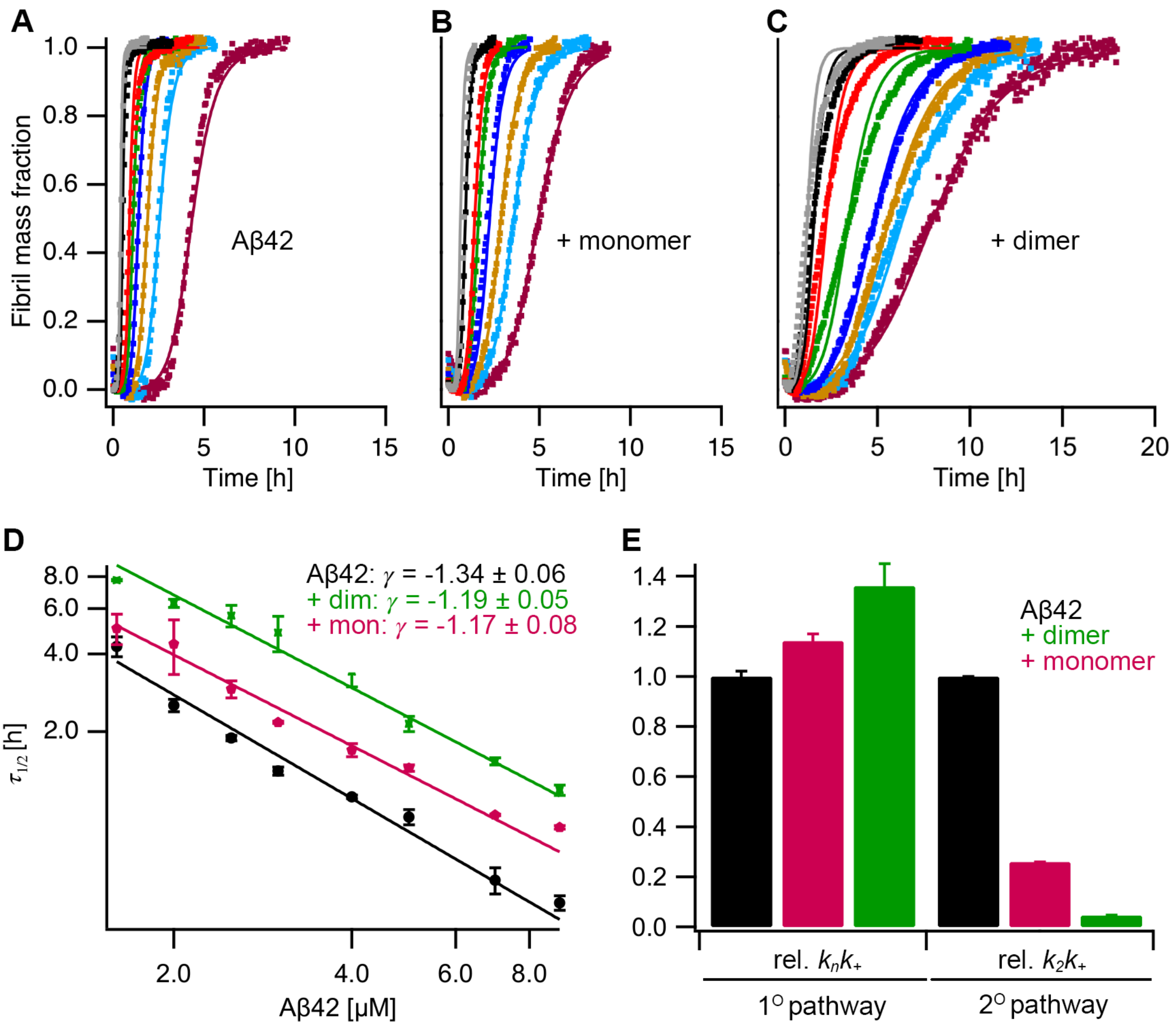

Supplementary Figure 6. Global fits of aggregation traces of Aβ42 in the presence and absence of rh Bri2 BRICHOS R221E species. (A-C) Global fits (solid lines) of aggregation traces (dots) with different Aβ42 concentrations from 1.6 (purple), 2.0 (light blue), 2.5 (yellow), 3.0 (blue), 4.0 (green), 5.0 (red), 7.0 (black) to 9 (grey) µM in the absence or in the presence of 0.9 µM rh Bri2 BRICHOS R221E species, where $\sqrt{k_n k_+}$ and $\sqrt{k_+ k_2}$ are constrained to the same value across all concentrations. (**A**) Aβ42 alone, $\sqrt{k_+ k_n}$ =6.32±0.13 M^{-1} s^{-1}, $\sqrt{k_+ k_2}$ =1.99×10^5±10 $M^{-3/2}$ s^{-1}, χ^2 =1.21. (**B**) Aβ42 with rh Bri2 BRICHOS R221E monomer, $\sqrt{k_+ k_n}$ =6.76±0.18 M^{-1} s^{-1}, $\sqrt{k_+ k_2}$ =1.01×10^5±40 $M^{-3/2}$ s^{-1}, χ^2 =1.50. (**C**) Aβ42 with rh Bri2 BRICHOS R221E dimer $\sqrt{k_+ k_n}$ =7.38 ± 0.57 M^{-1} s^{-1}, $\sqrt{k_+ k_2}$ =0.42×10^5±160 $M^{-3/2}$ s^{-1}, χ^2 =3.22. **(D)** Aβ42 in the absence (black) and presence of 0.9 µM rh Bri2 BRICHOS R221E monomers or dimers exhibit a similar dependence of the aggregation half-

time, $\tau_{1/2}$, on the initial peptide monomer concentration, described by the γ-exponent. **(E)** Global analysis from the data set in **A-C** revealed a dominant effect on, k_+k_2 related to secondary nucleation.

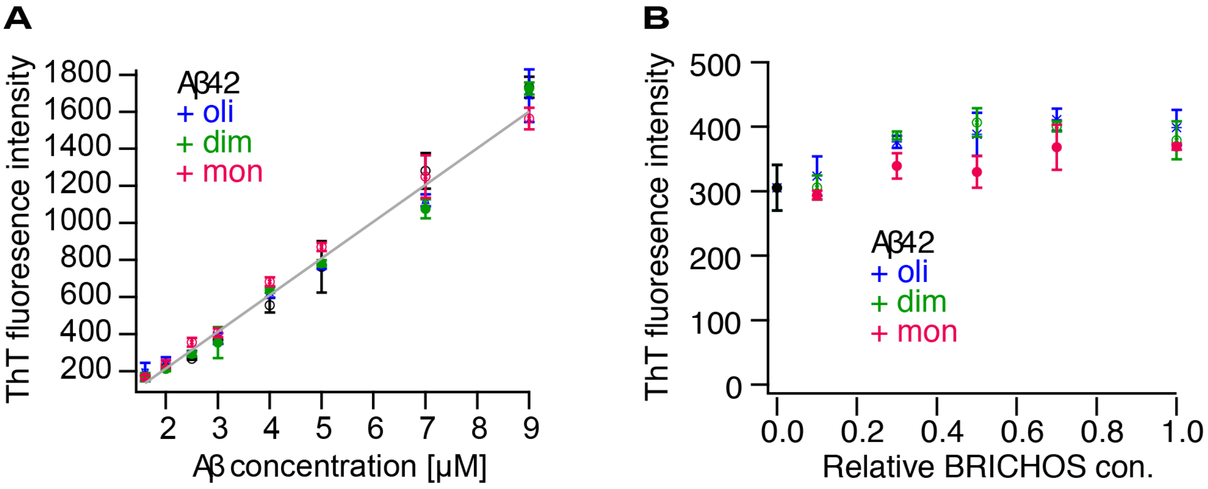

Supplementary Figure 7. Final ThT fluorescence intensity in the absence and presence of rh Bri2 BRICHOS R221E species. (**A**) Final ThT fluorescence intensity of Aβ42 under different starting concentrations from 1.6, 2.0, 2.5, 3.0, 4.0, 5.0, 7.0 to 9 µM in the absence (black) or in the presence of 0.9 µM rh Bri2 BRICHOS R221E monomer (red), dimer (green) and oligomer (blue). (**B**) Final ThT fluorescence intensity of 3 µM Aβ42 in the presence of rh Bri2 BRICHOS R221E monomer (red), dimer (green) and oligomer (blue) at concentrations: 0, 10, 30, 50, 70 or 100 molar percentage referred to monomeric subunits relative to Aβ42.

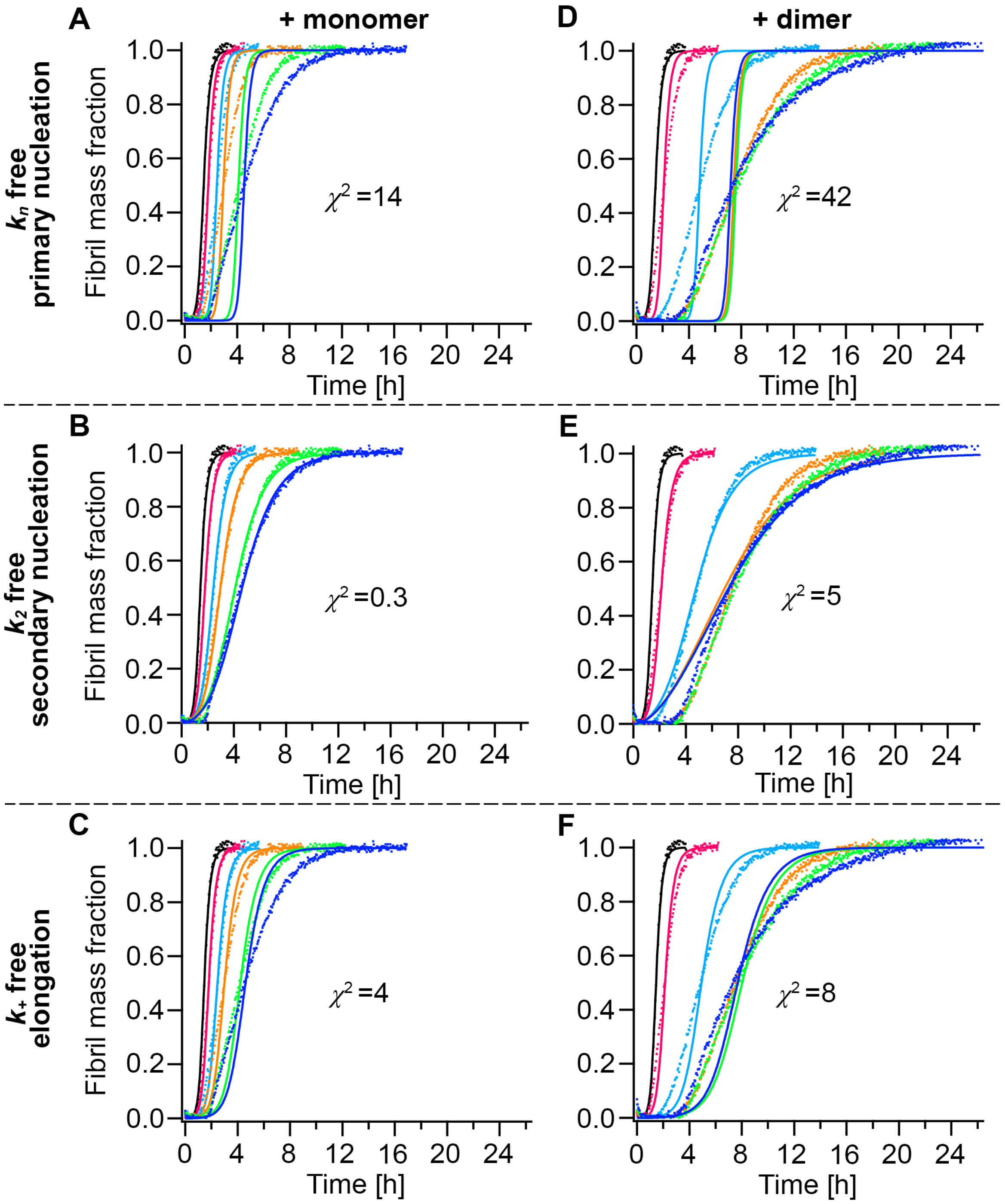

Supplementary Figure 8. Aggregation kinetics of Aβ42 in the presence of different concentrations of rh Bri2 BRICHOS R221E species. Aggregation kinetics of 3 µM Aβ42 in the presence of rh Bri2 BRICHOS R221E monomer (**A-C**) and dimer (**D-F**) at concentrations: 0 (black), 10 (purple), 30 (cyan), 50 (yellow), 70 (green) or 100 (blue) molar percentage referred to monomeric subunits relative to Aβ42. The global fits (solid lines) of the aggregation traces (dots) were constrained such that only one single rate constant is the free fitting parameter, indicated to the left of each row. χ^2 values describing the quality of the fits are shown in each figure.

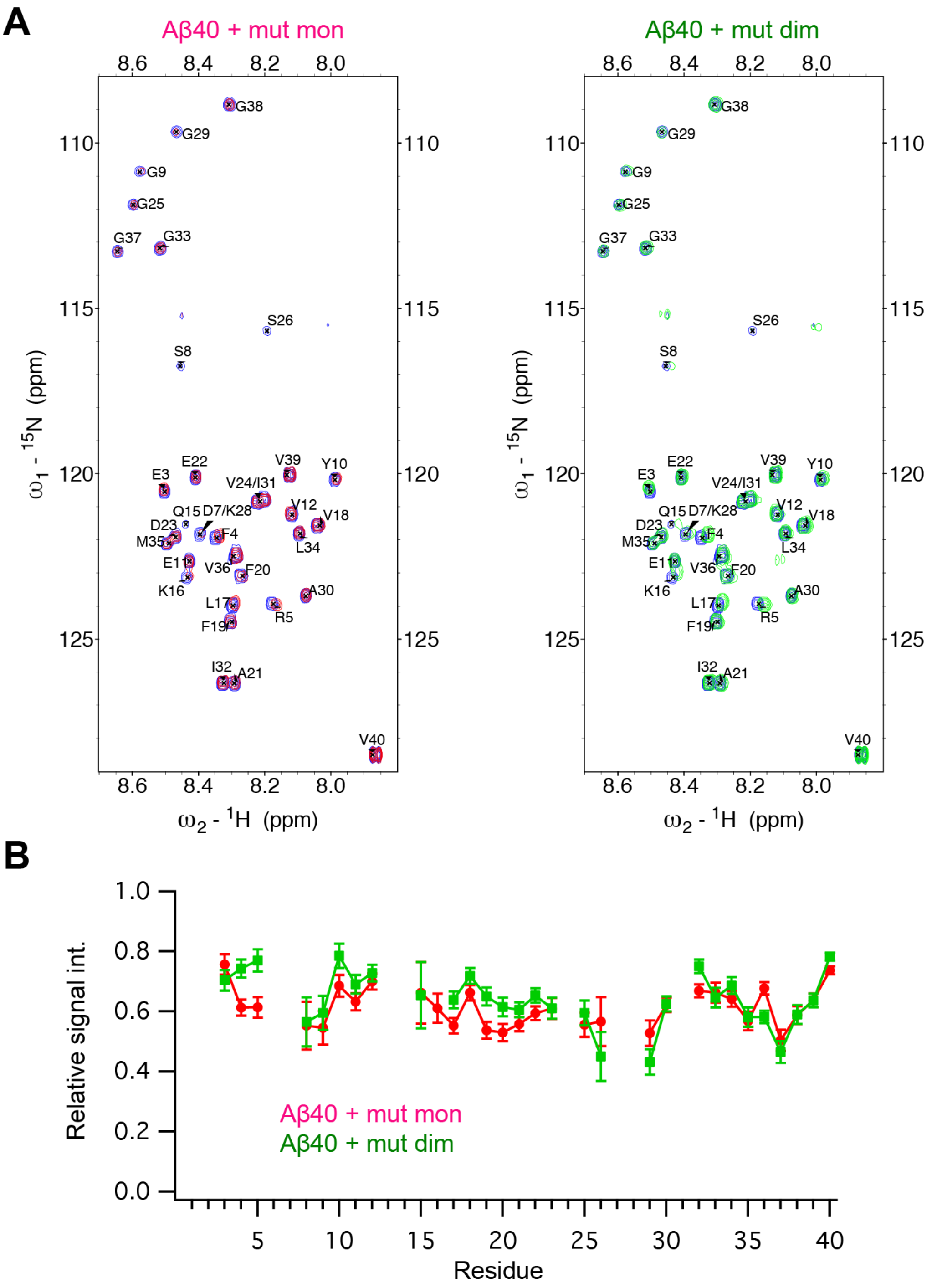

Supplementary Figure 9. HSQC spectrum of ^{15}N-Aβ$_{40}$ in the absence and presence of rh Bri2 BRICHOS monomer and dimer. (**A**) Overlay of ^{1}H-^{15}N HSQC spectra of 75 µM ^{15}N-Aβ$_{40}$ alone (blue) and in the presence of 75 µM Bri2 BRICHOS R221E monomer (red) or 58 µM dimer (green), recorded at 500 MHz and 8 °C. (**B**) Relative signal intensities of ^{1}H-^{15}N HSQC cross-peaks in the presence of 75 µM Bri2 BRICHOS R221E monomer (red) or 58 µM dimer (green). The error bars reflect the signal-to-noise ratio.

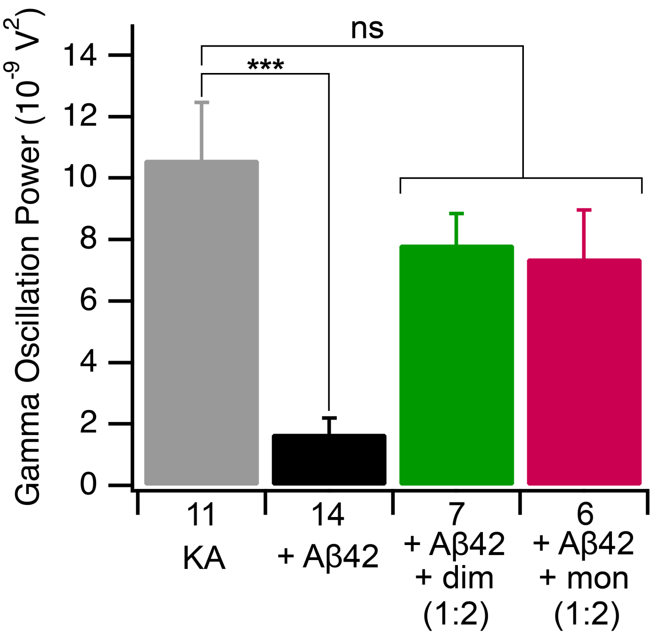

Supplementary Figure 10. Effects on Aβ42 toxicity in mouse hippocampal slices of different rh Bri2 BRICHOS R221E species. Summary histogram of mouse hippocampal γ oscillation power under control conditions (grey), after 15 min incubation with 50 nM Aβ42 (black), after 15 min incubation with 50 nM Aβ42 + 100 nM dimers (green) or 100 nM monomers (red). The numbers under the histograms denote the number of biological replicates, and the data are reported as means ± standard errors of the means. *** $p<0.001$; ns, no significant difference.
