# Supplementary material for: Augmentation of Bri2 molecular chaperone activity against amyloid-β reduces neurotoxicity in mouse hippocampus in vitro
Source: Commun Biol. 2020 Jan 20;3:32. doi: 10.1038/s42003-020-0757-z (PMC6971075; doi:10.1038/s42003-020-0757-z)
Supplement: Supplementary file 2 — Description of Additional Supplementary Files [file 42003_2020_757_MOESM2_ESM.pdf]

Descriptions of additional supplementary files.

Supplementary data file 1 contains raw data for Figure 2C and D and Figure 4B.

Supplementary data file 2 contains raw data for Figure 3A, B and C.

Supplementary data file 3 contains raw data for Figure 3D, E, and F.
