# Supplementary material for: Augmentation of Bri2 molecular chaperone activity against amyloid-β reduces neurotoxicity in mouse hippocampus in vitro
Source: Commun Biol. 2020 Jan 20;3:32. doi: 10.1038/s42003-020-0757-z (PMC6971075; doi:10.1038/s42003-020-0757-z)
Supplement: Supplementary file 6 — Reporting Summary [file 42003_2020_757_MOESM6_ESM.pdf]

nature research

Corresponding author(s): Jan Johansson

Last updated by author(s): Nov 13, 2019

Reporting Summary

Nature Research wishes to improve the reproducibility of the work that we publish. This form provides structure for consistency and transparency in reporting. For further information on Nature Research policies, see Authors & Referees and the Editorial Policy Checklist.

Statistics

For all statistical analyses, confirm that the following items are present in the figure legend, table legend, main text, or Methods section.

n/a	Confirmed	
☐	☒	The exact sample size (n) for each experimental group/condition, given as a discrete number and unit of measurement
☐	☒	A statement on whether measurements were taken from distinct samples or whether the same sample was measured repeatedly
☐	☒	The statistical test(s) used AND whether they are one- or two-sided<br>*Only common tests should be described solely by name; describe more complex techniques in the Methods section.*
☒	☐	A description of all covariates tested
☒	☐	A description of any assumptions or corrections, such as tests of normality and adjustment for multiple comparisons
☒	☐	A full description of the statistical parameters including central tendency (e.g. means) or other basic estimates (e.g. regression coefficient) AND variation (e.g. standard deviation) or associated estimates of uncertainty (e.g. confidence intervals)
☒	☐	For null hypothesis testing, the test statistic (e.g. F, t, r) with confidence intervals, effect sizes, degrees of freedom and P value noted<br>*Give P values as exact values whenever suitable.*
☒	☐	For Bayesian analysis, information on the choice of priors and Markov chain Monte Carlo settings
☒	☐	For hierarchical and complex designs, identification of the appropriate level for tests and full reporting of outcomes
☒	☐	Estimates of effect sizes (e.g. Cohen's d, Pearson's r), indicating how they were calculated

Our web collection on statistics for biologists contains articles on many of the points above.

Software and code

Policy information about availability of computer code

Data collection	Not relevant
Data analysis	Not relevant

For manuscripts utilizing custom algorithms or software that are central to the research but not yet described in published literature, software must be made available to editors/reviewers. We strongly encourage code deposition in a community repository (e.g. GitHub). See the Nature Research guidelines for submitting code & software for further information.

Data

Policy information about availability of data

All manuscripts must include a data availability statement. This statement should provide the following information, where applicable:

- Accession codes, unique identifiers, or web links for publicly available datasets
- A list of figures that have associated raw data
- A description of any restrictions on data availability

All data and materials related to this paper are available from J.J. (janne.johansson@ki.se).

Field-specific reporting

Please select the one below that is the best fit for your research. If you are not sure, read the appropriate sections before making your selection.

☒ Life sciences ☐ Behavioural & social sciences ☐ Ecological, evolutionary & environmental sciences

For a reference copy of the document with all sections, see nature.com/documents/nr-reporting-summary-flat.pdf

Life sciences study design

All studies must disclose on these points even when the disclosure is negative.

Sample size	Sample size as conventionally used for these types of experiments.
Data exclusions	No data excluded.
Replication	The number of biological replicates are given in the experimental sections.
Randomization	Not relevant for the present experiments.
Blinding	Blinding not possible due to experimental design.

Reporting for specific materials, systems and methods

We require information from authors about some types of materials, experimental systems and methods used in many studies. Here, indicate whether each material, system or method listed is relevant to your study. If you are not sure if a list item applies to your research, read the appropriate section before selecting a response.

Materials & experimental systems

n/a	Involved in the study	
☐	☒	Antibodies
☒	☐	Eukaryotic cell lines
☒	☐	Palaeontology
☐	☒	Animals and other organisms
☒	☐	Human research participants
☒	☐	Clinical data

Methods

n/a	Involved in the study	
☒	☐	ChIP-seq
☒	☐	Flow cytometry
☒	☐	MRI-based neuroimaging

Antibodies

Antibodies used	Rabbit anti AU1 antibody (Abcam, UK), goat anti-Bri2 BRICHOS antibody.
Validation	The goat anti-Bri2 BRICHOS antibody is not a commercial antibody, but has been used in previously studies against recombinant human Bri2 BRICHOS proteins, as referenced in the manuscript. The rabbit anti AU1 antibody is specific for the AU1 tag, as indicated by the manufacturer's description and also has been used in previous studies, also referenced.

Animals and other organisms

Policy information about studies involving animals; ARRIVE guidelines recommended for reporting animal research

Laboratory animals	C57BL/6 mice of either sex (postnatal days 14-23, supplied from Charles River, Germany).
Wild animals	Not relevant.
Field-collected samples	Not relevant.
Ethics oversight	Norra Stockholm's Djurförsöksetiska Nämnd (dnr N45/13).

Note that full information on the approval of the study protocol must also be provided in the manuscript.
